# Supplementary material for: Differential contribution of PBP occupancy and efflux on the effectiveness of β-lactams at their target site in clinical isolates of Neisseria gonorrhoeae
Source: PLoS Pathog. 2024 Dec 31;20(12):e1012783. doi: 10.1371/journal.ppat.1012783 (PMC11729944; doi:10.1371/journal.ppat.1012783)
Supplement: S1 Table — a N. gonorrhoeae strains ATCC 19424 and ATCC 49226; clinical strains NG 3, NG 7, NG 12, NG 14, NG 19, NG 20, NG 21 from Hospital Universitario Son Espases (Spain) and NG 22 from Hospital Clínic de Barcelona (Spain); and WHO reference strains NCTC 13820 (WHO X), NCTC 13821 (WHO Y) and NCTC 13822 (WHO Z). MLST, multilocus sequence typing; NG-MAST, Neisseria gonorrhoeae multiantigen sequence typing; NG-STAR, Neisseria gonorrhoeae Sequence Typing for Antimicrobial Resistance; ST, sequence type; WT, wild type. New NG-STAR profiles ST5411 and ST5569 were described. b The sequences and complete genomes for the N. gonorrhoeae ATCC strains 19424 and 49226, were obtained from the ATCC (American Type Culture Collection) genome portal. c The sequences for the N. gonorrhoeae clinical strains, were obtained from whole genome sequencing (WGS). d The sequences and complete genomes for the N. gonorrhoeae NCTC strains 13820 (WHO X), NCTC 13821 (WHO Y) and NCTC 13822 (WHO Z), were obtained from the BioProject PRJEB14020. (PDF) [file ppat.1012783.s001.pdf]

S1 Table. Genetic characteristics of *Neisseria gonorrhoeae* strains ATCC 19424 and 49226, clinical strains and WHO reference strains X, Y and Z.

**Strain**[a]	**MLST**[a]	**NG-MAST v2.0**[a]	**NG-STAR**[a]	***ponA* (PBP1)**	***penA* (PBP2)**	***penA* Allele**	***dacB* (PBP3)**	***pbpG* (PBP4)**	***mtrR* promoter**	***mtrR***	***porB***	***23S* rRNA**	***rpsJ***	***gyrA***	***parC***
ATCC 19424[b]	ST11248	ST266	-	WT	D345a	XV	T252S, H278Q, S285A, I364M	I307M	WT	WT	WT (1a)	WT	WT	WT	WT
ATCC 49226[b]	ST11075	ST1572	ST1	WT	F504L	XXII	T252S, H278Q, S285A, I364M	I307M	WT	A39T	WT (1a)	WT	V57M	WT	WT
NG3[c]	ST7827	ST22418	ST38	L421P	A501V; F504L; P551S	XIII	T252S, H278Q, I364M	I307M	-35A Del	G45D	G120K, A121D (1b)	WT	V57M	S91F, D95G	D86N
NG7[c]	ST9363	ST20729	ST417	WT	F504L	II	T252S, H278Q, S285A, I364M	I307M	*N. meningitidis*-like promoter	WT	G120K, A121N (1b)	C2611T	V57M	WT	WT
NG12[c]	ST1901	ST1407	*ST5569*	L421P	I312M; V316T; D345a; A501V; F504L; N512Y; G545S	XXXIV mosaic	T252S, H278Q, I364M	I307M	-35A Del	WT	G120K, A121N (1b)	WT	V57M	S91F, D95G	D86N
NG14[c]	ST1901	ST4951	*ST5569*	L421P	I312M; V316T; D345a; A501V; F504L; N512Y; G545S	XXXIV mosaic	T252S, H278Q, I364M	I307M	-35A Del	WT	G120K, A121N (1b)	WT	V57M	S91F, D95G	D86N
NG19[c]	ST7822	ST21392	*ST5411*	L421P	F504L; P551S	V	T252S, H278Q, I364M	I307M	*N. meningitidis*-like promoter	A39T	G120K, A121N (1b)	WT	V57M	S91F, D95A	S87R
NG20[c]	ST1580	ST649	ST192	WT	F504L	II	T252S, H278Q, I364M	I307M	WT	G45D	A121S (1b)	A2059G	V57M	WT	WT
NG21[c]	ST7827	ST22418	ST38	L421P	A501V; F504L;	XIII	T252S, H278Q,	I307M	-35A Del	G45D	G120K, A121D	WT	V57M	S91F, D95G	D86N

					P551S		I364M				(1b)				
NG22[c]	ST7827	ST2318	ST38	L421P	A501V, F504L, P551S	XIII	T252S, H278Q, I364M	I307M	-35A Del	G45D	G120K, A121D (1b)	WT	V57M	S91F, D95G	D86N
WHO X[d]	ST7363	ST4220	ST226	L421P	A311V, I312M, V316P, D345a, T483S F504L, N512Y, G545S	XXXVII mosaic	T252S, H278Q, I364M	I307M	-35A Del	WT	G120K, A121D (1b)	WT	V57M	S91F, D95N	S87R, S88P
WHO Y[d]	ST1901	ST1407	ST16	L421P	I312M, V316P, D345a, A501P, F504L, N512Y G545S	XLII mosaic	T252S, H278Q, I364M	I307M	-35A Del	WT	G120K, A121N (1b)	WT	V57M	S91F, D95G	S87R
WHO Z[d]	ST7363	ST4015	ST227	L421P	A311V; I312M; V316T; D345a; T483S; F504L; N512Y; G545S	LXIV mosaic	T252S, H278Q, I364M	I307M	-35 A to C	WT	G120K, A121D (1b)	WT	V57M	S91F, D95N	S87R, S88P

[a] *N. gonorrhoeae* strains ATCC 19424 and ATCC 49226; clinical strains NG 3, NG 7, NG 12, NG 14, NG 19, NG 20, NG 21 from Hospital Universitario Son Espases (Spain) and NG 22 from Hospital Clínic de Barcelona (Spain); and WHO reference strains NCTC 13820 (WHO X), NCTC 13821 (WHO Y) and NCTC 13822 (WHO Z). MLST, multilocus sequence typing; NG-MAST, *Neisseria gonorrhoeae* multiantigen sequence typing; NG-STAR, *Neisseria gonorrhoeae* Sequence Typing for Antimicrobial Resistance; ST, sequence type; WT, wild type. New NG-STAR profiles ST5411 and ST5569 were described. [b] The sequences and complete genomes for the *N. gonorrhoeae* ATCC strains 19424

and 49226, were obtained from the ATCC (American Type Culture Collection) genome portal. [c] The sequences for the *N. gonorrhoeae* clinical strains, were obtained from whole genome sequencing (WGS). [d] The sequences and complete genomes for the *N. gonorrhoeae* NCTC strains 13820 (WHO X), NCTC 13821 (WHO Y) and NCTC 13822 (WHO Z), were obtained from the BioProject PRJEB14020.
