# Supplementary material for: Differential contribution of PBP occupancy and efflux on the effectiveness of β-lactams at their target site in clinical isolates of Neisseria gonorrhoeae
Source: PLoS Pathog. 2024 Dec 31;20(12):e1012783. doi: 10.1371/journal.ppat.1012783 (PMC11729944; doi:10.1371/journal.ppat.1012783)
Supplement: S2 Table — a N. gonorrhoeae strains ATCC 19424 and ATCC 49226; clinical strains NG 3, NG 7, NG 12, NG 14, NG 19, NG 20, NG 21 from Hospital Universitario Son Espases (Spain) and NG 22 from Hospital Clínic de Barcelona (Spain); and WHO reference strains NCTC 13820 (WHO X), NCTC 13821 (WHO Y) and NCTC 13822 (WHO Z). b PBP, penicillin-binding proteins. c This table shows the concentration of β-lactam required to inhibit 50% of Bocillin FL compared to a control with no drug. The mean values from three experiments are presented. The abbreviations used are as follows: ETP for ertapenem, CFM for cefixime, CTX for cefotaxime, CRO for ceftriaxone, CAZ for ceftazidime, TOL for ceftolozane, PIP for piperacillin, AVI for avibactam, TZ for tazobactam, CAZ/AVI for ceftazidime/avibactam, TOL/TZ for ceftolozane/tazobactam, and PIP/TZ for piperacillin/tazobactam. d When the primary PBP target was not inhibited by the regular concentrations (0.001 to 0.125 mg/L or 0.016 to 2 mg/L), an extended range of 1 to 512 mg/L was used. For CAZ/AVI, TOL/TZ, and PIP/TZ, a fixed concentration of the BLIs avibactam or tazobactam at 4 mg/L was used. (PDF) [file ppat.1012783.s002.pdf]

S2 Table. PBP IC_{50} ± SD of β-lactam antibiotics and BLIs in *N. gonorrhoeae* strains.

Strain[a]	Mean IC_{50} ± SD of the indicated drug (mg/L)[c]						
	**PBP**[b]	**ETP**	**CFM**	**CTX**	**CRO**	**CAZ**	**AVI**
ATCC 19424	PBP1	0.11 ± 0.05	0.38 ± 0.04	0.25 ± 0.09	0.19 ± 0.003	1.26 ± 0.48	> 512
	PBP2	0.003 ± 0.001	0.002 ± 0.001	0.001 ± 0.0005	0.002 ± 0.001	0.006 ± 0.001	33 ± 16
	PBP3	< 0.001	> 2	0.35 ± 0.05	0.006 ± 0.001	> 2	3.04 ± 0.37
ATCC 49226	PBP1	0.08 ± 0.02	0.47 ± 0.18	0.29 ± 0.16	0.18 ± 0.02	1.62 ± 0.02	> 512
	PBP2	0.006 ± 0.001	0.003 ± 0.001	0.003 ± 0.001	0.003 ± 0.001	0.01 ± 0.004	380 ± 59
	PBP3	< 0.001	> 2	0.388 ± 0.253	0.011 ± 0.001	> 2	1.84 ± 0.01
NG 3	PBP1	0.05 ± 0.01	1.85 ± 0.48	0.56 ± 0.18	0.37 ± 0.003	> 2	> 512
	PBP2	0.003 ± 0.001	0.02 ± 0.004	0.01 ± 0.004	0.02 ± 0.004	0.1 ± 0.003	437 ± 63
	PBP3	<0.001	1.67 ± 0.08	0.26 ± 0.09	0.02 ± 0.008	> 2	1.70 ± 0.15
NG 7	PBP1	0.08 ± 0.03	0.15 ± 0.03	0.52 ± 0.13	0.19 ± 0.02	1.35 ± 0.77	> 512
	PBP2	0.008 ± 0.004	0.004 ± 0.002	0.006 ± 0.001	0.005 ± 0.001	0.02 ± 0.002	302 ± 92
	PBP3	< 0.001	> 2	0.6 ± 0.18	0.02 ± 0.005	> 2	3.01 ± 0.44
NG 12	PBP1	0.06 ± 0.02	0.80 ± 0.01	0.57 ± 0.23	0.29 ± 0.12	> 2	> 512
	PBP2	0.02 ± 0.01	0.05 ± 0.003	0.03 ± 0.003	0.02 ± 0.005	0.13 ± 0.05	> 512
	PBP3	< 0.001	> 2	0.39 ± 0.08	0.02 ± 0.002	> 2	2.36 ± 0.74
NG 14[d]	PBP1	0.08 ± 0.01	1.06 ± 0.28	0.77 ± 0.07	0.20 ± 0.16	> 2	> 512
	PBP2	0.03 ± 0.01	0.04 ± 0.01	0.03 ± 0.01	0.01 ± 0.003	0.10 ± 0.01	464 ± 71
	PBP3	< 0.001	1.87 ± 0.17	0.55 ± 0.20	0.02 ± 0.009	> 2	2.95 ± 0.04
NG 19	PBP1	0.05 ± 0.007	1.36 ± 0.07	0.51 ± 0.16	0.43 ± 0.31	> 2	> 512
	PBP2	0.004 ± 0.001	0.002 ± 0.001	0.005 ± 0.001	0.006 ± 0.001	0.02 ± 0.008	372 ± 33
	PBP3	< 0.001	> 2	0.40 ± 0.02	0.02 ± 0.004	> 2	2.36 ± 0.55
NG 20	PBP1	0.08 ± 0.03	0.14 ± 0.03	0.26 ± 0.05	0.08 ± 0.02	1.57 ± 0.16	> 512
	PBP2	0.005 ± 0.001	0.004 ± 0.001	0.004 ± 0.002	0.003 ± 0.001	0.02 ± 0.002	306 ± 134
	PBP3	< 0.001	> 2	0.71 ± 0.11	0.006 ± 0.002	1.67 ± 0.17	2.14 ± 0.45
NG 21	PBP1	0.07 ± 0.01	0.76 ± 0.11	0.57 ± 0.23	0.24 ± 0.04	> 2	> 512
	PBP2	0.011 ± 0.002	0.02 ± 0.005	0.02 ± 0.005	0.01 ± 0.002	0.07 ± 0.02	385 ± 7

	PBP3	< 0.001	> 2	0.43 ± 0.10	0.02 ± 0.003	> 2	1.98 ± 0.07
NG 22	PBP1	0.05 ± 0.01	0.82 ± 0.11	0.83 ± 0.43	0.32 ± 0.01	> 2	> 512
	PBP2	0.005 ± 0.002	0.01 ± 0.002	0.02 ± 0.005	0.02 ± 0.002	0.08 ± 0.02	343 ± 121
	PBP3	< 0.001	> 2	0.55 ± 0.18	0.02 ± 0.001	> 2	2.63 ± 0.15
WHO X[d]	PBP1	0.08 ± 0.007	1.65 ± 0.31	0.63 ± 0.19	0.19 ± 0.02	3.53 ± 0.21	> 512
	PBP2	0.03 ± 0.002	4.86 ± 2.68	4.76 ± 1.64	0.86 ± 0.19	4.44 ± 1.79	> 512
	PBP3	< 0.001	1.74 ± 0.31	0.38 ± 0.03	0.01 ± 0.002	3.07 ± 0.31	2.49 ± 0.07
WHO Y[d]	PBP1	0.08 ± 0.01	1.07 ± 0.42	0.41 ± 0.10	0.23 ± 0.08	3.76 ± 1.56	> 512
	PBP2	0.003 ± 0.001	11 ± 1.12	1.98 ± 0.71	0.51 ± 0.25	15 ± 4	24 ± 2
	PBP3	< 0.001	2.11 ± 0.51	0.27 ± 0.07	0.02 ± 0.005	3.78 ± 1.92	1.96 ± 0.75
WHO Z[d]	PBP1	0.08 ± 0.02	2.11 ± 1.09	0.55 ± 0.17	0.33 ± 0.015	4.67 ± 1.73	> 512
	PBP2	0.01 ± 0.002	1.33 ± 0.11	0.44 ± 0.006	0.14 ± 0.02	0.60 ± 0.23	> 512
	PBP3	< 0.001	1.67 ± 0.08	0.34 ± 0.004	0.01 ± 0.005	3.37 ± 0.39	3.29 ± 0.23

Table S2 (Continued).

**Strain[a]**	Mean IC_{50} ± SD of the indicated drug (mg/L)[c]						
	**PBP[b]**	**CAZ/AVI[e]**	**TOL**	**PIP**	**TZ**	**TOL/TZ[e]**	**PIP/TZ[e]**
ATCC 19424	PBP1	> 2	> 2	> 2	7.93 ± 5.80	> 2	1.94 ± 0.753
	PBP2	0.007 ± 0.004	0.01 ± 0.002	0.002 ± 0.001	2.33 ± 0.05	< 0.001	< 0.001
	PBP3	< 0.001	> 2	0.02 ± 0.005	2.97 ± 0.19	< 0.001	< 0.001
ATCC49226	PBP1	2.07 ± 0.47	> 2	> 2	20 ± 2	> 2	> 2
	PBP2	0.01 ± 0.005	0.06 ± 0.003	0.01 ± 0.002	1.28 ± 0.06	< 0.001	< 0.001
	PBP3	< 0.001	> 2	0.03 ± 0.02	1.58 ± 0.23	< 0.001	< 0.001
NG 3	PBP1	1.69 ± 0.19	> 2	1.94 ± 0.97	25 ± 4	> 2	> 2
	PBP2	0.04 ± 0.01	0.15 ± 0.05	0.01 ± 0.005	1.15 ± 0.43	< 0.001	< 0.001
	PBP3	< 0.001	> 2	0.02 ± 0.004	2.10 ± 0.67	< 0.001	< 0.001
NG 7	PBP1	1.91 ± 0.22	> 2	1.73 ± 0.18	17 ± 8	> 2	> 2
	PBP2	0.03 ± 0.005	0.08 ± 0.03	0.02 ± 0.008	1.24 ± 0.01	< 0.001	< 0.001

	PBP3	0.05 ± 0.04	> 2	0.03 ± 0.003	1.82 ± 0.09	< 0.001	< 0.001
NG 12	PBP1	> 2	> 2	> 2	42 ± 14	> 2	> 2
	PBP2	0.15 ± 0.04	1.43 ± 0.14	0.02 ± 0.006	6.91 ± 0.15	0.51 ± 0.20	0.004 ± 0.001
	PBP3	< 0.001	> 2	0.03 ± 0.006	1.63 ± 0.01	< 0.001	< 0.001
NG 14	PBP1	> 2	26 ± 4	> 2	33 ± 11	> 2	> 2
	PBP2	0.16 ± 0.05	2.38 ± 0.59	0.02 ± 0.003	7.27 ± 0.37	1.06 ± 0.48	0.003 ± 0.000
	PBP3	< 0.001	35 ± 9	0.025 ± 0.004	1.72 ± 0.18	< 0.001	< 0.001
NG 19	PBP1	> 2	> 2	> 2	32 ± 11	> 2	> 2
	PBP2	0.03 ± 0.01	0.17 ± 0.03	0.02 ± 0.004	0.87 ± 0.11	< 0.001	< 0.001
	PBP3	< 0.001	> 2	0.040 ± 0.002	2.08 ± 0.71	< 0.001	< 0.001
NG 20	PBP1	1.81 ± 0.71	> 2	1.62 ± 0.38	12 ± 1	> 2	1.05 ± 0.001
	PBP2	0.018 ± 0.005	0.07 ± 0.03	0.02 ± 0.001	1.28 ± 0.08	< 0.001	< 0.001
	PBP3	< 0.001	> 2	0.04 ± 0.002	1.71 ± 0.01	< 0.001	< 0.001
NG 21	PBP1	1.89 ± 0.10	> 2	> 2	42 ± 24	> 2	> 2
	PBP2	0.07 ± 0.02	0.21 ± 0.002	0.02 ± 0.002	0.95 ± 0.04	< 0.001	< 0.001
	PBP3	< 0.001	> 2	0.03 ± 0.007	2.23 ± 0.62	< 0.001	< 0.001
NG 22	PBP1	> 2	> 2	> 2	29 ± 1	> 2	> 2
	PBP2	0.07 ± 0.01	0.20 ± 0.01	0.01 ± 0.004	1.42 ± 0.20	< 0.001	< 0.001
	PBP3	< 0.001	> 2	0.02 ± 0.002	2.04 ± 0.66	< 0.001	< 0.001
WHO X[d]	PBP1	2.80 ± 0.33	20 ± 2	> 2	26 ± 2	15 ± 6	> 2
	PBP2	2.12 ± 0.60	39 ± 13	0.04 ± 0.004	36 ± 19	52 ± 7	0.02 ± 0.002
	PBP3	< 0.001	33 ± 10	0.03 ± 0.005	1.72 ± 0.11	< 0.001	< 0.001
WHO Y[d]	PBP1	5.26 ± 0.04	23 ± 4	> 2	25 ± 1	24 ± 4	> 2
	PBP2	26 ± 2	> 128	0.03 ± 0.004	2.50 ± 0.09	< 0.001	< 0.001
	PBP3	< 0.001	25 ± 3	0.03 ± 0.003	1.73 ± 0.11	< 0.001	< 0.001
WHO Z[d]	PBP1	4.95 ± 0.29	18 ± 7	> 2	24 ± 0.44	19 ± 8	> 2
	PBP2	0.63 ± 0.04	6.85 ± 0.50	0.03 ± 0.004	26 ± 0.41	6.92 ± 0.21	0.02 ± 0.001
	PBP3	< 0.001	24 ± 0.3	0.03 ± 0.001	1.29 ± 0.47	< 0.001	< 0.001

[a] *N. gonorrhoeae* strains ATCC 19424 and ATCC 49226; clinical strains NG 3, NG 7, NG 12, NG 14, NG 19, NG 20, NG 21 from Hospital Universitario Son Espases (Spain) and NG 22 from Hospital Clínic de Barcelona (Spain); and WHO reference strains NCTC 13820 (WHO X), NCTC 13821 (WHO Y) and NCTC 13822 (WHO Z). [b] PBP, penicillin-binding proteins. [c] This table shows the concentration of β-lactam required to inhibit 50% of Bocillin FL compared to a control with no drug. The mean values from three experiments are presented. The abbreviations used are as follows: ETP for ertapenem, CFM for cefixime, CTX for cefotaxime, CRO for ceftriaxone, CAZ for ceftazidime, TOL for ceftolozane, PIP for piperacillin, AVI for avibactam, TZ for tazobactam, CAZ/AVI for ceftazidime/avibactam, TOL/TZ for ceftolozane/tazobactam, and PIP/TZ for piperacillin/tazobactam. [d] When the primary PBP target was not inhibited by the regular concentrations (0.001 to 0.125 mg/L or 0.016 to 2 mg/L), an extended range of 1 to 512 mg/L was used. For CAZ/AVI, TOL/TZ, and PIP/TZ, a fixed concentration of the BLIs avibactam or tazobactam at 4 mg/L was used.
