# Supplementary material for: Differential contribution of PBP occupancy and efflux on the effectiveness of β-lactams at their target site in clinical isolates of Neisseria gonorrhoeae
Source: PLoS Pathog. 2024 Dec 31;20(12):e1012783. doi: 10.1371/journal.ppat.1012783 (PMC11729944; doi:10.1371/journal.ppat.1012783)
Supplement: S3 Table — a Pearson correlation coefficient. b To calculate an estimated ratio, all MIC values less than 0.002 were converted to 0.001. (PDF) [file ppat.1012783.s003.pdf]

S3 Table. MIC and PBP2 IC_{50} ratio.

Strain	PBP2 variant	MIC/PBP2_IC_{50} ratio											
		**ETP**	**CFM**	**CTX**	**CRO**	**CAZ**	**TOL**	**PIP**	**AVI**	**TZ**	**CAZ/AVI**	**TOL/TZ**[b]	**PIP/TZ**[b]
NG19424	XXII	1.40	2.35	4.55	1.10	2.47	2.02	1.02	3.88	0.05	1.15	2.00	2.00
NG49226	XV	1.40	4.90	2.30	1.45	2.19	4.45	9.30	0.67	0.78	4.04	2.00	62.50
NG3	XIII	1.56	9.30	6.08	3.47	13.18	19.98	17.58	0.75	5.65	6.62	2.00	500.00
NG7	II	0.36	6.89	2.46	0.83	1.38	5.93	13.37	0.85	1.61	2.37	2.00	31.25
NG12	XXXIV	1.25	2.49	8.65	6.30	7.66	5.60	6.21	0.50	2.31	3.34	3.92	64.55
NG14	XXXIV	1.19	1.42	8.22	5.75	9.94	0.84	1.79	0.55	2.28	3.05	1.89	42.94
NG19	V	0.96	3.40	5.69	1.38	4.56	0.72	1.91	0.69	1.17	3.76	2.00	2.00
NG20	II	1.69	4.25	7.22	1.31	6.47	6.76	6.13	0.84	1.56	7.07	2.00	2.00
NG21	XIII	1.27	1.97	8.87	3.41	5.05	0.81	2.39	0.59	1.73	11.63	2.00	500.00
NG22	XIII	1.46	3.55	7.81	4.86	13.62	5.05	5.78	0.66	4.21	6.92	1.95	125.00
WHO X	XXXVII	1.20	0.82	1.68	2.31	3.60	1.64	3.06	0.50	1.25	7.63	1.24	11.80
WHO Z	LXIV	1.18	1.50	9.02	3.64	6.67	2.33	4.60	0.50	1.78	0.30	4.63	12.28
WHO Y	XLII	0.61	0.48	2.02	1.45	1.05	1.00	1.22	1.35	1.60	0.61	2.00	31.25
PBP selectivity		PBP2<br>PBP3	PBP2	PBP2	PBP2	PBP2	PBP2	PBP2<br>PBP3	PBP3	PBP2<br>PBP3	PBP3	PBP2<br>PBP3	PBP2<br>PBP3
ρ^a		0.99	0.94	0.93	0.97	0.85	0.98	*0.22*	0.86	0.97	*0.69*	0.94	*0.23*

[a] Pearson correlation coefficient. [b] To calculate an estimated ratio, all MIC values less than 0.002 were converted to 0.001.
