# Supplementary material for: Differential contribution of PBP occupancy and efflux on the effectiveness of β-lactams at their target site in clinical isolates of Neisseria gonorrhoeae
Source: PLoS Pathog. 2024 Dec 31;20(12):e1012783. doi: 10.1371/journal.ppat.1012783 (PMC11729944; doi:10.1371/journal.ppat.1012783)
Supplement: S4 Table — a The following N. gonorrhoeae strains were studied: ATCC 19424 and ATCC 49226; clinical strains NG 3, NG 7, NG 12, NG 14, NG 19, NG 20, NG 21 from Hospital Universitario Son Espases (Spain) and NG 22 from Hospital Clínic de Barcelona (Spain); and WHO reference strains NCTC 13820 (WHO X), NCTC 13821 (WHO Y) and NCTC 13822 (WHO Z). b Broth microdilution MICs were performed following CLSI guidelines [1]. The antibiotics/EPI tested were ertapenem (ETP), cefixime (CFM), cefotaxime (CTX), ceftriaxone (CRO), ceftazidime (CAZ), ceftolozane (TOL), piperacillin (PIP), avibactam (AVI), tazobactam (TZ), ceftazidime/avibactam (CAZ/AVI), ceftolozane/tazobactam (TOL/TZ), piperacillin/tazobactam (PIP/TZ), azithromycin (AZT) carbonyl cyanide 3-chlorophenylhydrazone (CCCP) and PAβN (Phe-Arg-β-naphthylamide). c PIP/TZ, TOL/TZ and CAZ/AVI MICs were conducted using a fixed concentration of 4 mg/L BLI avibactam or tazobactam. c MICs were performed with a fixed concentration of the EPI CCCP (0.1 mg/L) and PAβN (25 mg/L). d FC: MIC fold change after the addition of PaβN or CCCP. Bold numbers indicate a ≥4-fold MIC reduction when combined with EPI. (PDF) [file ppat.1012783.s004.pdf]

**S4 Table.** Ratio between FA 1090 MIC and *penA* FA 1090 transformants.

| | Ratio MIC FA1090/MIC transformants FA 1090 | | | | | | | |
|---|---|---|---|---|---|---|---|---|
| **Strain** | **penG** | **ETP** | **CFM** | **CTX** | **CRO** | **CAZ/AVI** | **TOL/TZ** | **PIP/TZ** |
| FA 1090 | 1 | 1 | 1 | 1 | 1 | 1 | 1 | 1 |
| FA 1090 $penA_{NG3}$ | 2 | 2 | 2 | 8 | 2 | 4 | 1 | 1 |
| FA 1090 $penA_{NG12}$ | 2 | 6 | 8 | 16 | 2 | 8 | 2 | 1 |
| FA 1090 $penA_{NG14}$ | 2 | 6 | 8 | 16 | 4 | 8 | 1 | 1 |
| FA 1090 $penA_{NG21}$ | 2 | 1 | 2 | 8 | 2 | 4 | 1 | 1 |
| FA 1090 $penA_{NG22}$ | 2 | 1 | 2 | 4 | 2 | 2 | 1 | 1 |
| FA 1090 $penA_{WHOX}$ | 8 | 8 | 250 | 500 | 500 | 125 | 1000 | 1 |
| FA 1090 $penA_{WHOY}$ | 4 | 1 | 125 | 500 | 250 | 250 | 1 | 1 |
| FA 1090 $penA_{WHOZ}$ | 8 | 4 | 125 | 500 | 125 | 31 | 500 | 1 |
