# Supplementary material for: Differential contribution of PBP occupancy and efflux on the effectiveness of β-lactams at their target site in clinical isolates of Neisseria gonorrhoeae
Source: PLoS Pathog. 2024 Dec 31;20(12):e1012783. doi: 10.1371/journal.ppat.1012783 (PMC11729944; doi:10.1371/journal.ppat.1012783)
Supplement: S1 Fig — *CFM, 0.25–32 mg/L; CTX, 0.125–16 mg/L; CRO, 0.016–2 mg/L; CAZ, 0.25–32 mg/L; TOL, 1–128 mg/L; PIP, 0.016–2 mg/L; AVI, 2–256 mg/L; TZ, 1–128 mg/L; CAZ/AVI, 0.25–32 mg/L (avibactam 4 mg/L fixed concentration); TOL/TZ, 1–128 mg/L (tazobactam 4 mg/L fixed concentration). PBP-binding assay gels for N. gonorrhoeae strains ATCC 19424, ATCC 49226; clinical strains NG 3, NG 7, NG 12, NG 14, NG 19, NG 20, NG 21 and NG 22; WHO reference strains NCTC 13820 (WHO X), NCTC 13821 (WHO Y) and NCTC 13822 (WHO Z). 1 Drugs tested were ertapenem (ETP), cefixime (CFM), cefotaxime (CTX), ceftriaxone (CRO), ceftazidime (CAZ), ceftolozane (TOL), piperacillin (PIP), avibactam (AVI), tazobactam (TZ), ceftazidime/avibactam (CAZ/AVI), ceftolozane/tazobactam (TOL/TZ), and piperacillin/tazobactam (PIP/TZ). The antibiotic-bound PBP-containing membrane preparations were label with 25 μM Bocillin FLTM. Labeled PBPs were separated by SDS-PAGE and detected using a fluorimager. The global range of concentrations tested was 0.001 to 512 mg/L. (PDF) [file ppat.1012783.s006.pdf]

S1 Figure. Penicillin-binding protein occupancy dataset for 7 β-lactams, 2 β-lactamase inhibitors and combinations of 3 β-lactams/β-lactamase inhibitors in *Neisseria gonorrhoeae* strains ATCC 19424, ATCC 49226; clinical strains NG 3, NG 7, NG 12, NG 14, NG 19, NG 20, NG 21 and NG 22; WHO reference strains X, Y and WHO Z, at a global analyzed concentration range of 0.001 to 512 mg/L.

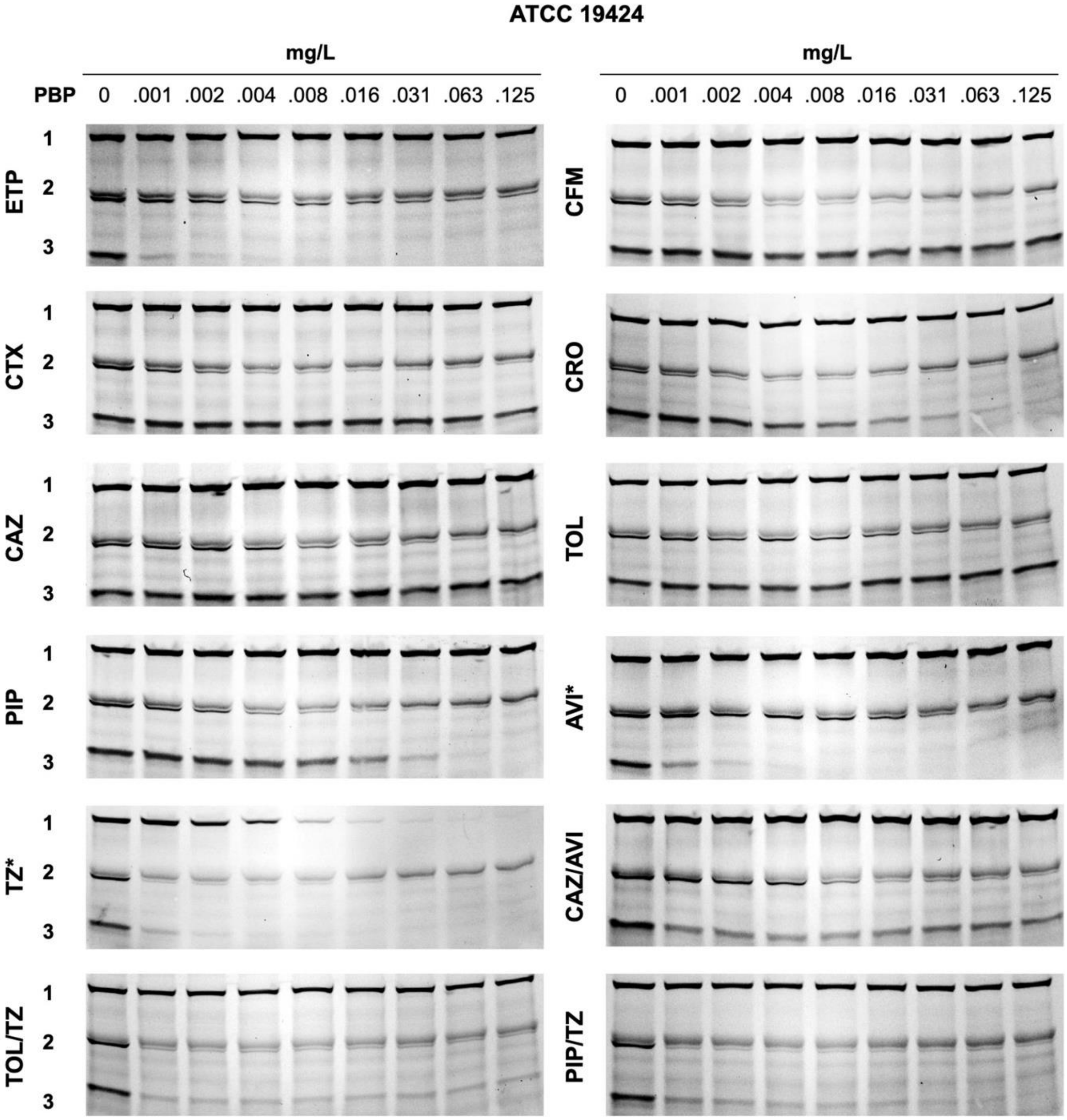

*AVI, 4-512 mg/L; TZ, 4-512 mg/L.

Figure S1. Continued.

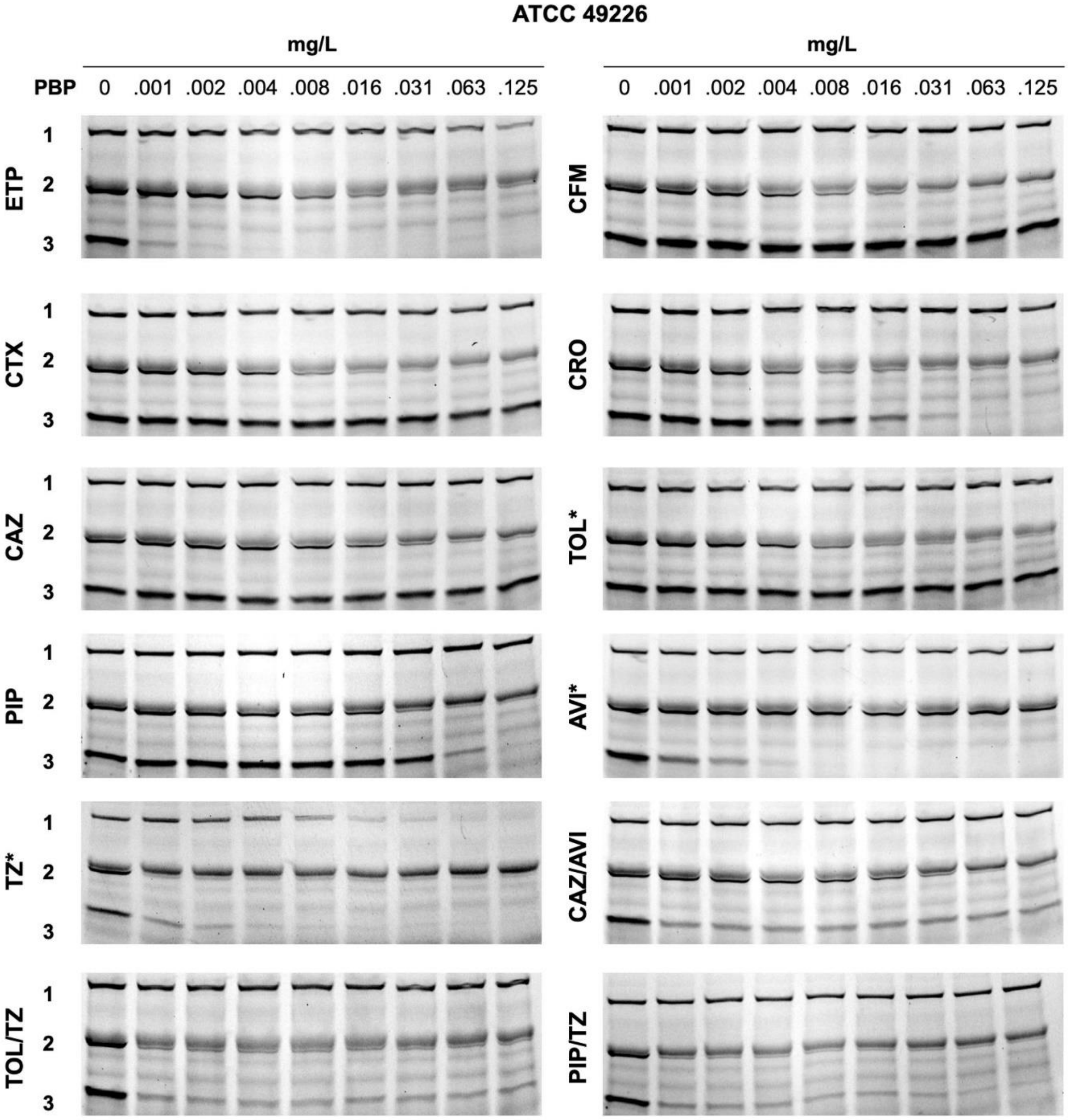

*TOL, 0.016-2 mg/L; AVI, 2-256 mg/L; TZ, 2-256 mg/L.

Figure S1. Continued.

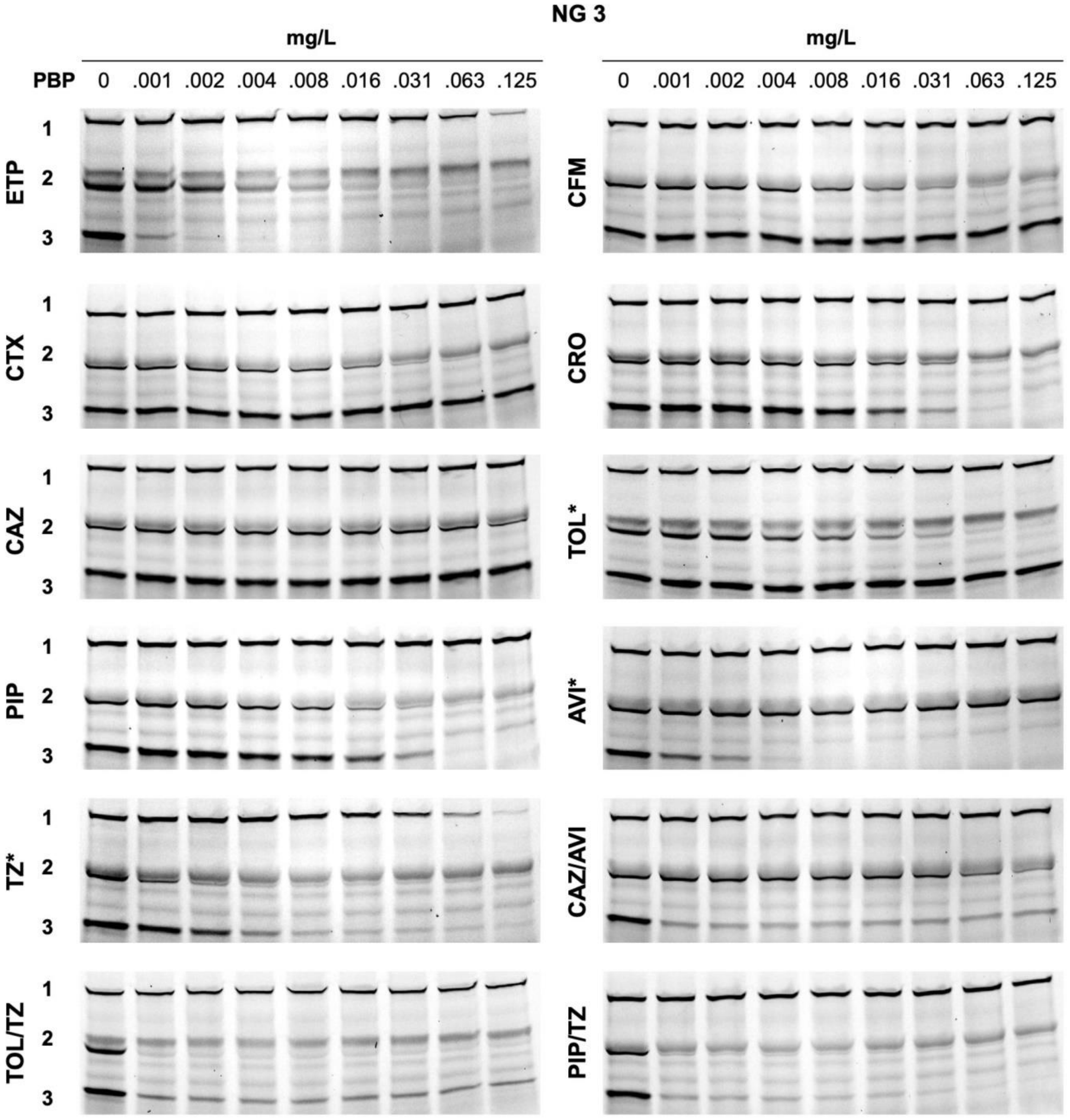

*TOL, 0.016-2 mg/L; AVI, 2-256 mg/L; TZ, 1-128 mg/L.

Figure S1. Continued.

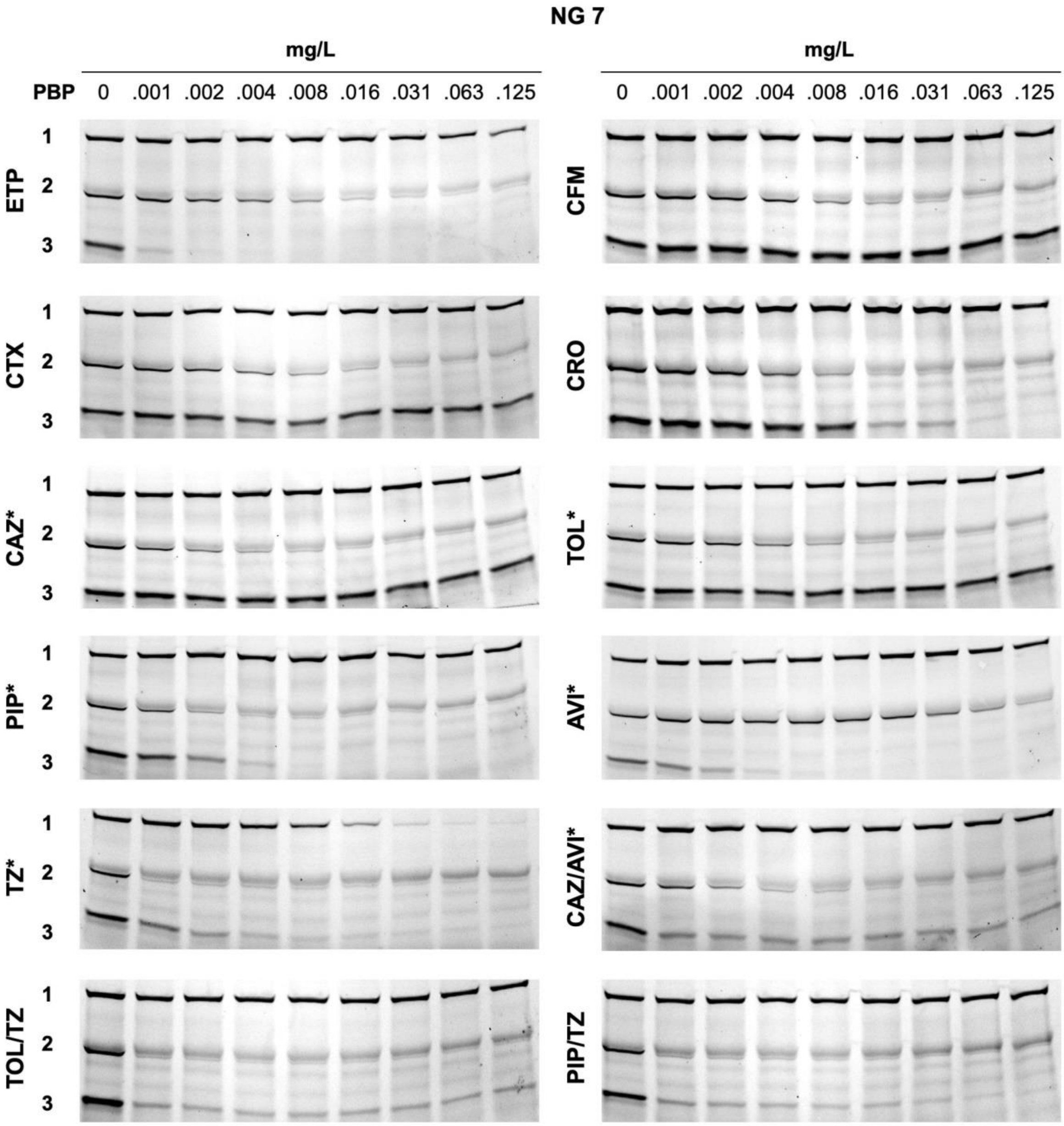

*CAZ, 0.016-2 mg/L; TOL, 0.016-2 mg/L; PIP, 0.016-2 mg/L; AVI, 2-256 mg/L; TZ, 2-256 mg/L; CAZ/AVI, 0.016-2 mg/L (avibactam 4 mg/L fixed concentration).

Figure S1. Continued.

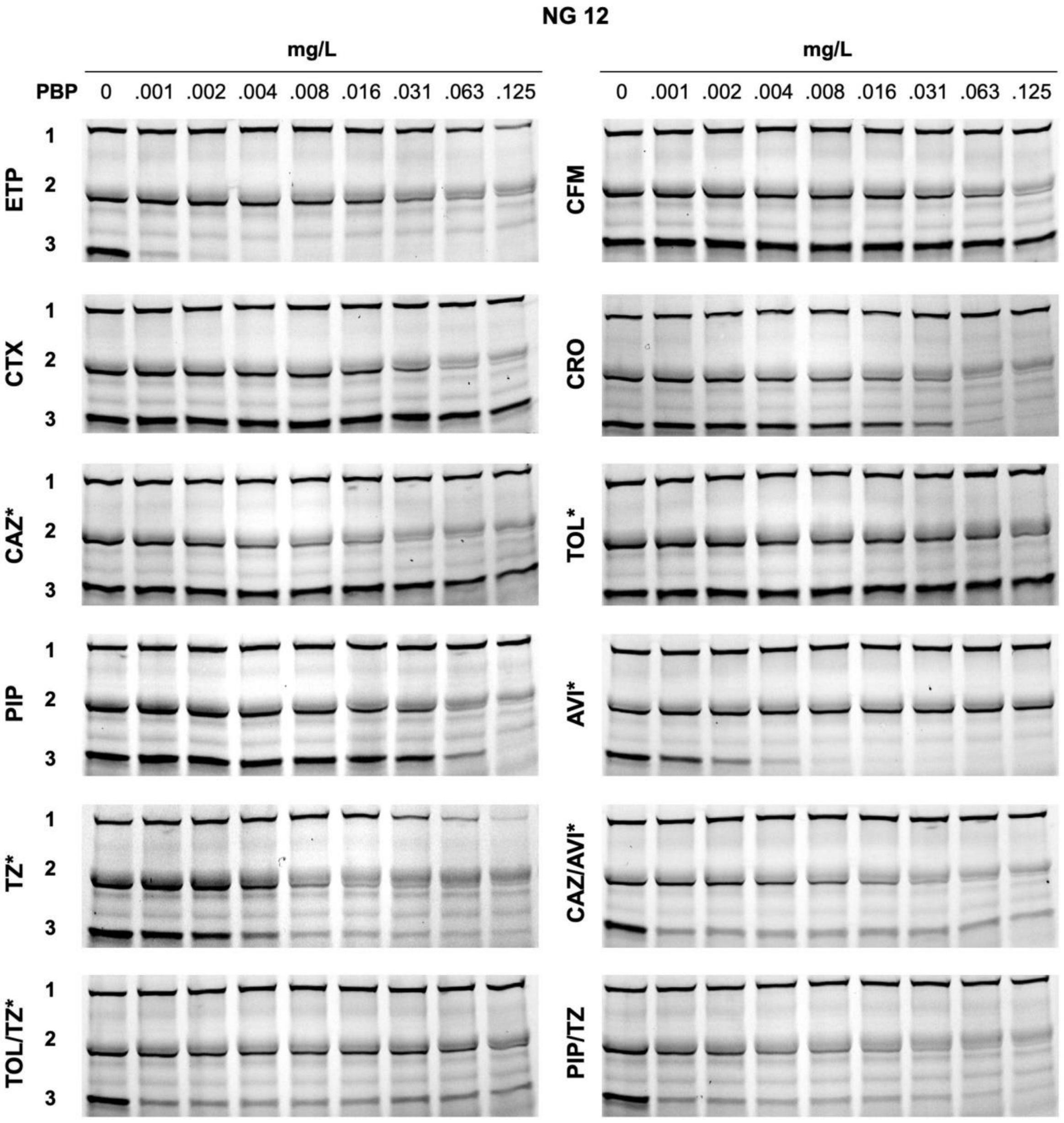

*CAZ, 0.016-2 mg/L; TOL, 0.016-2 mg/L; AVI, 2-256 mg/L; TZ, 1-128 mg/L; CAZ/AVI, 0.016-2 mg/L (avibactam 4 mg/L fixed concentration); TOL/TZ, 0.016-2 mg/L (tazobactam 4 mg/L fixed concentration).

Figure S1. Continued.

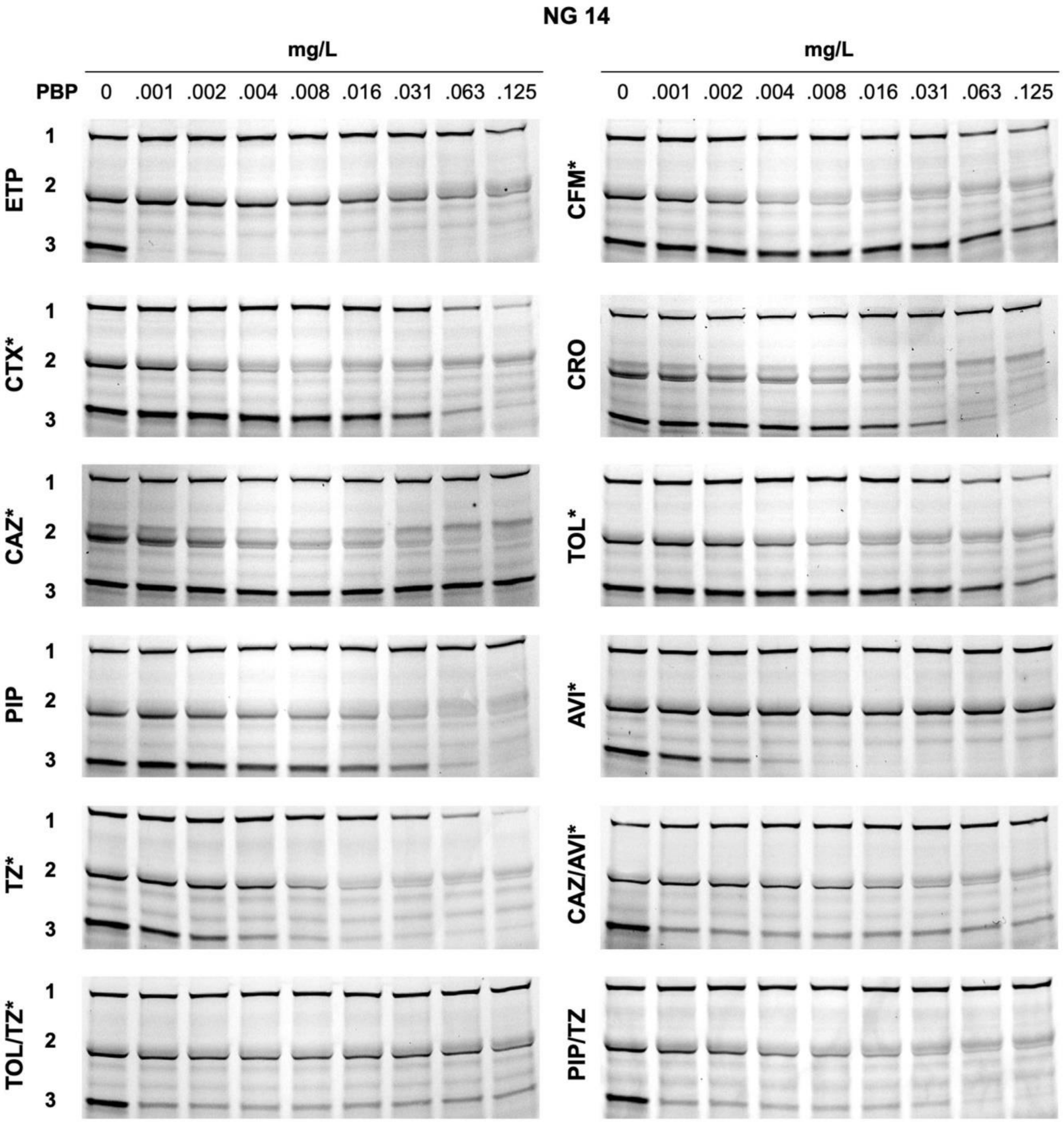

*CFM, 0.016-2 mg/L; CTX, 0.016-2 mg/L; CAZ, 0.016-2 mg/L; TOL, 0.5-64 mg/L; AVI, 2-256 mg/L; TZ, 1-128 mg/L; CAZ/AVI, 0.016-2 mg/L (avibactam 4 mg/L fixed concentration); TOL/TZ, 0.016-2 mg/L (tazobactam 4 mg/L fixed concentration).

Figure S1. Continued.

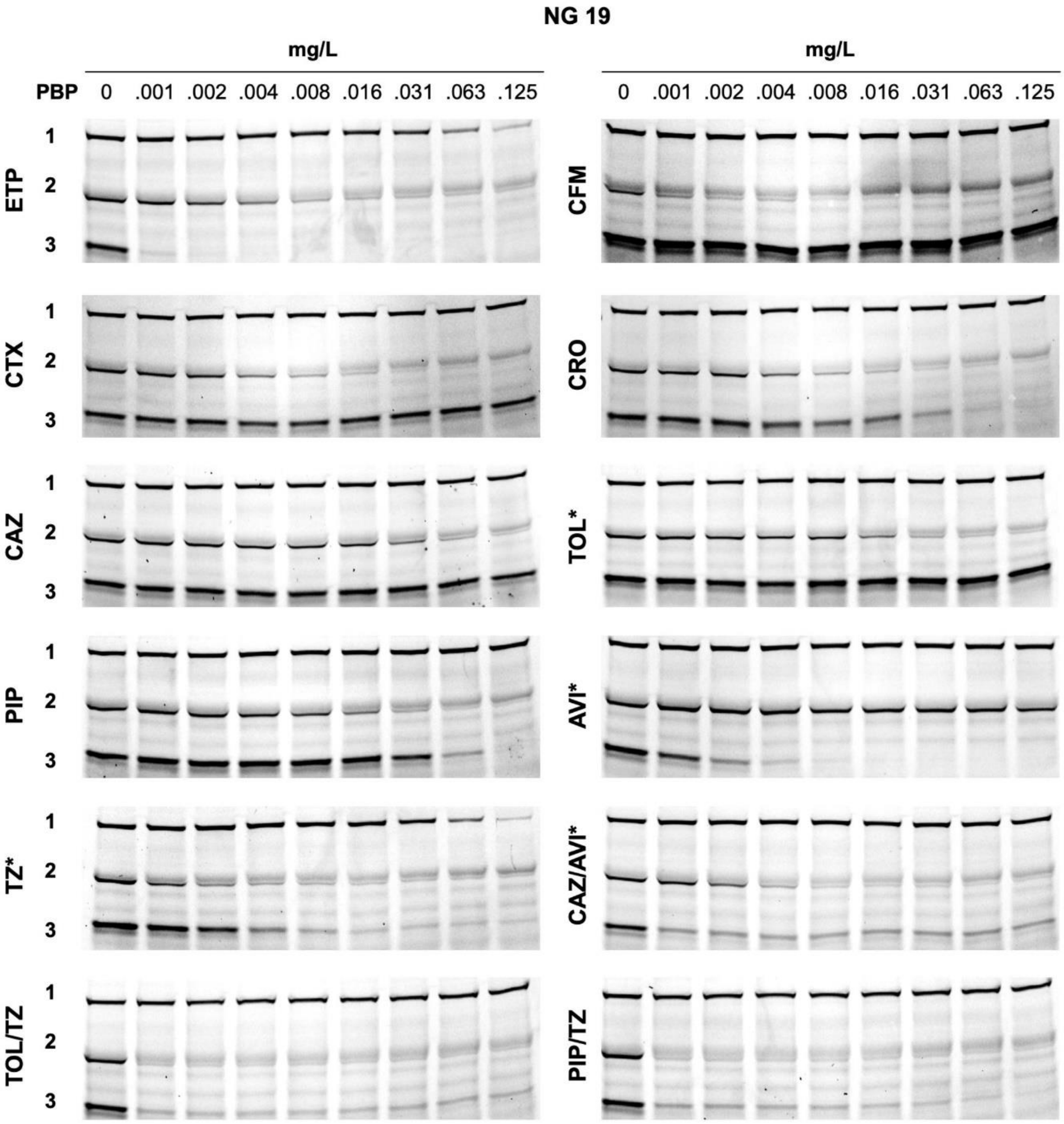

*TOL, 0.016-2 mg/L; AVI, 2-256 mg/L; TZ, 1-128 mg/L; CAZ/AVI, 0.016-2 mg/L (avibactam 4 mg/L fixed concentration).

Figure S1. Continued.

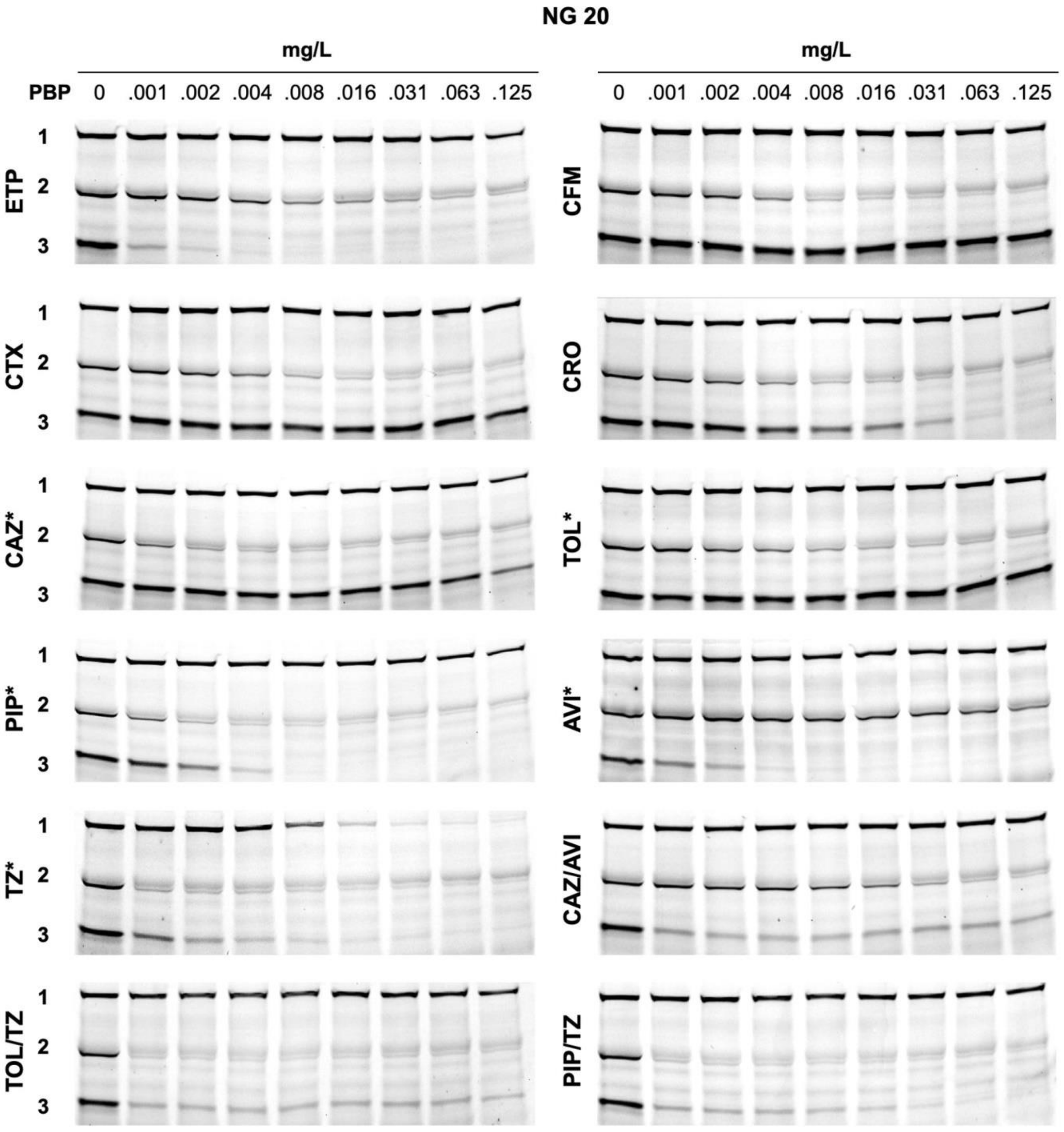

*CAZ, 0.016-2 mg/L; TOL, 0.016-2 mg/L; PIP, 0.016-2 mg/L; AVI, 2-256 mg/L; TZ, 2-256 mg/L.

Figure S1. Continued.

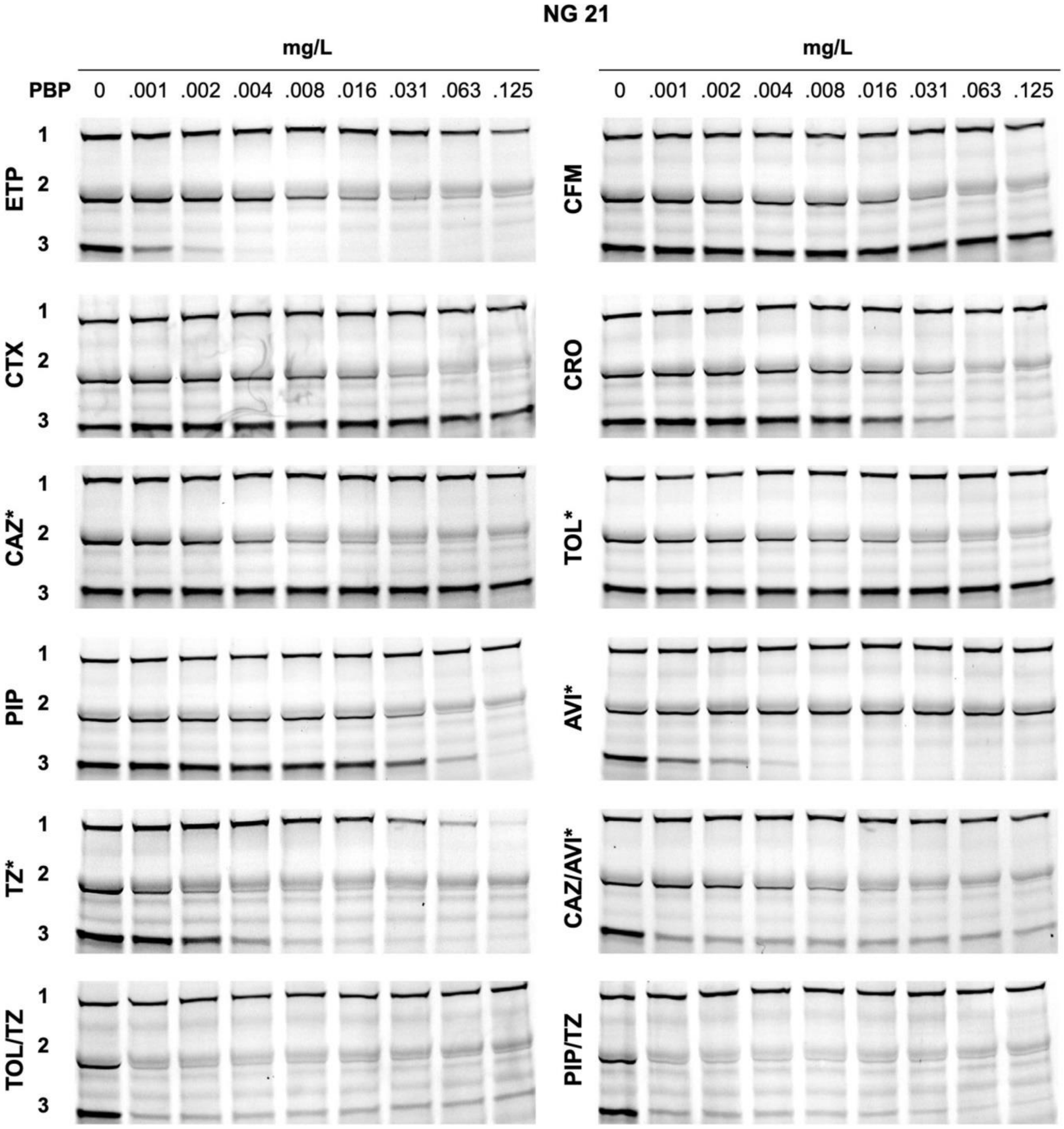

*CAZ, 0.016-2 mg/L; TOL, 0.016-2 mg/L; AVI, 2-256 mg/L; TZ, 1-128 mg/L; CAZ/AVI, 0.016-2 mg/L (avibactam 4 mg/L fixed concentration).

Figure S1. Continued.

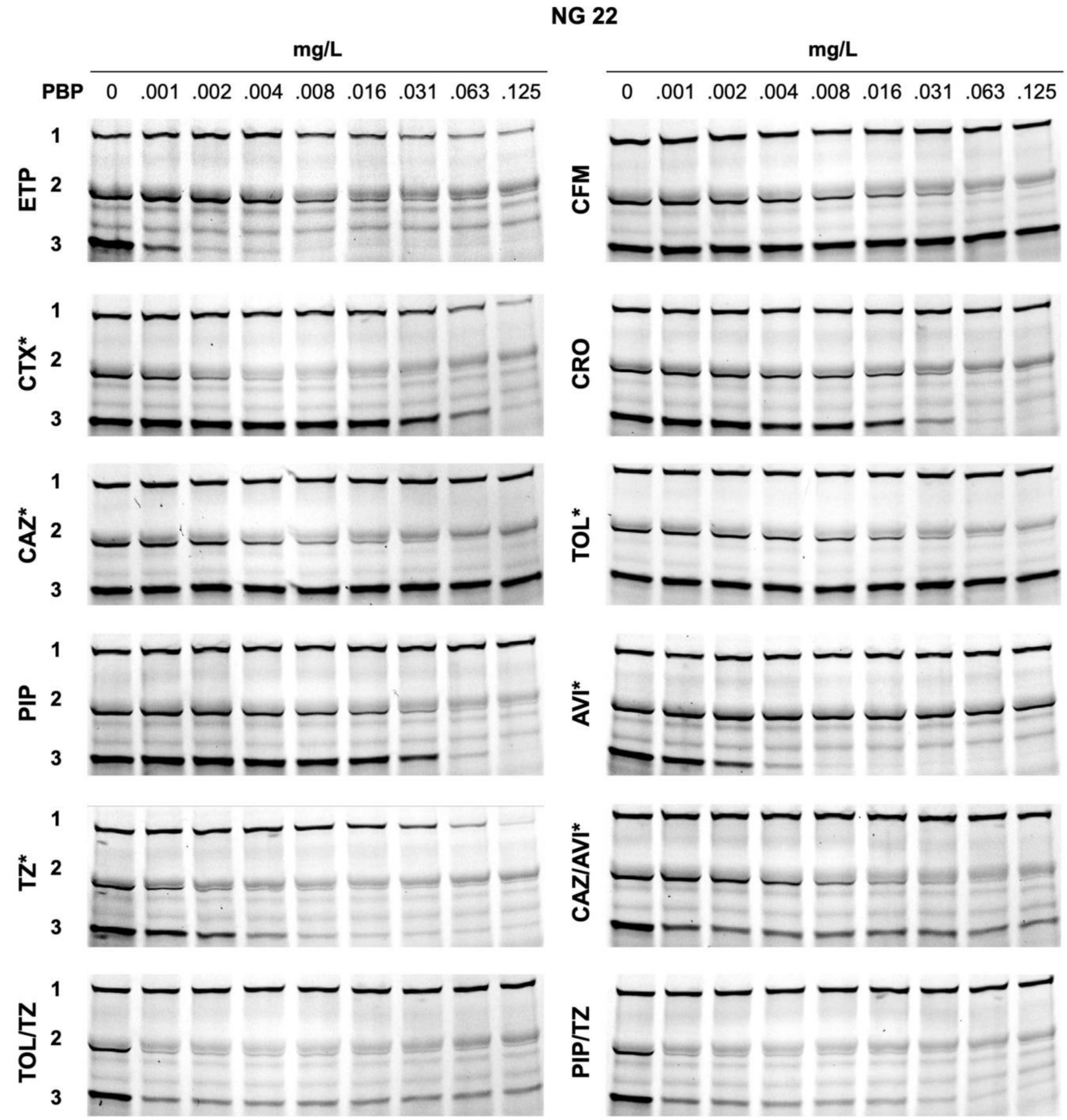

*CTX, 0.016-2 mg/L; CAZ, 0.016-2 mg/L; TOL, 0.016-2 mg/L; AVI, 2-256 mg/L; TZ, 1-128 mg/L; CAZ/AVI, 0.016-2 mg/L (avibactam 4 mg/L fixed concentration).

Figure S1. Continued.

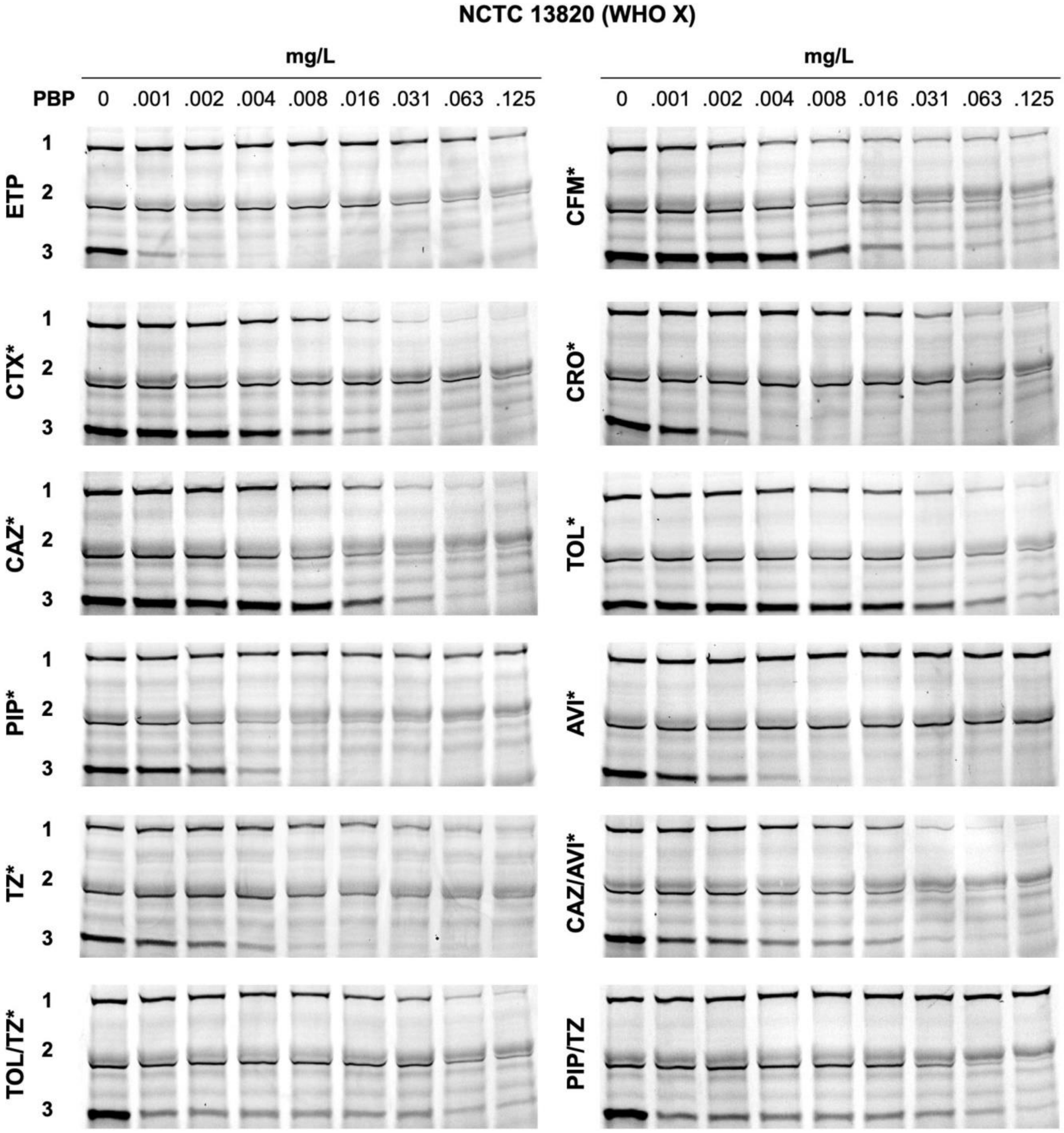

*CFM, 0.5-64 mg/L; CTX, 0.125-16 mg/L; CRO, 0.016-2 mg/L; CAZ, 0.5-64 mg/L; TOL, 1-128 mg/L; PIP, 0.016-2 mg/L; AVI, 2-256 mg/L; TZ, 1-128 mg/L; CAZ/AVI, 0.5-64 mg/L (avibactam 4 mg/L fixed concentration); TOL/TZ, 1-128 mg/L (tazobactam 4 mg/L fixed concentration).

Figure S1. Continued.

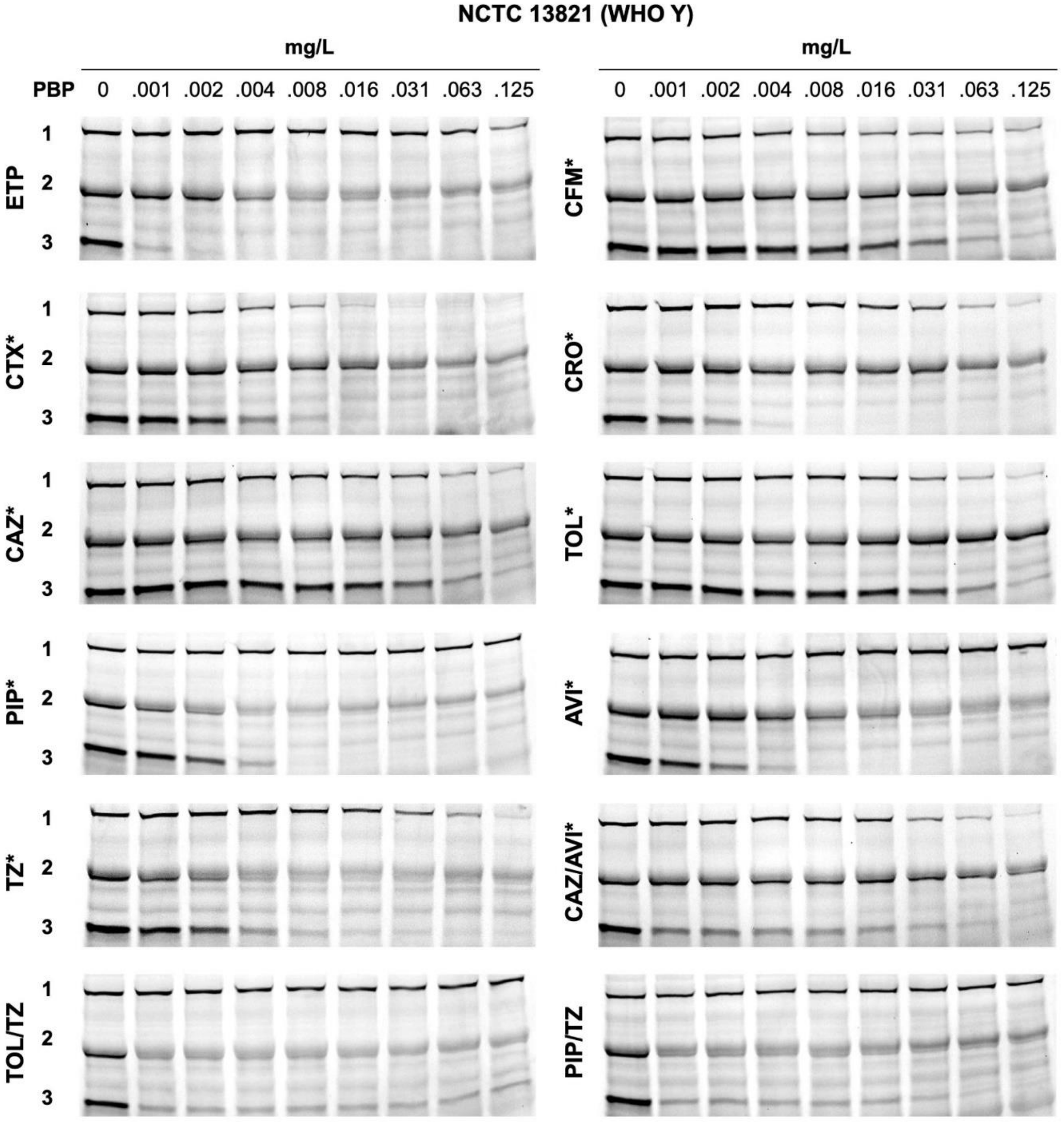

*CFM, 0.125-16 mg/L; CTX, 0.125-16 mg/L; CRO, 0.016-2 mg/L; CAZ, 0.125-16 mg/L; TOL, 1-128 mg/L; PIP, 0.016-2 mg/L; AVI, 2-256 mg/L; TZ, 1-128 mg/L; CAZ/AVI, 0.25-32 mg/L (avibactam 4 mg/L fixed concentration).

Figure S1. Continued.

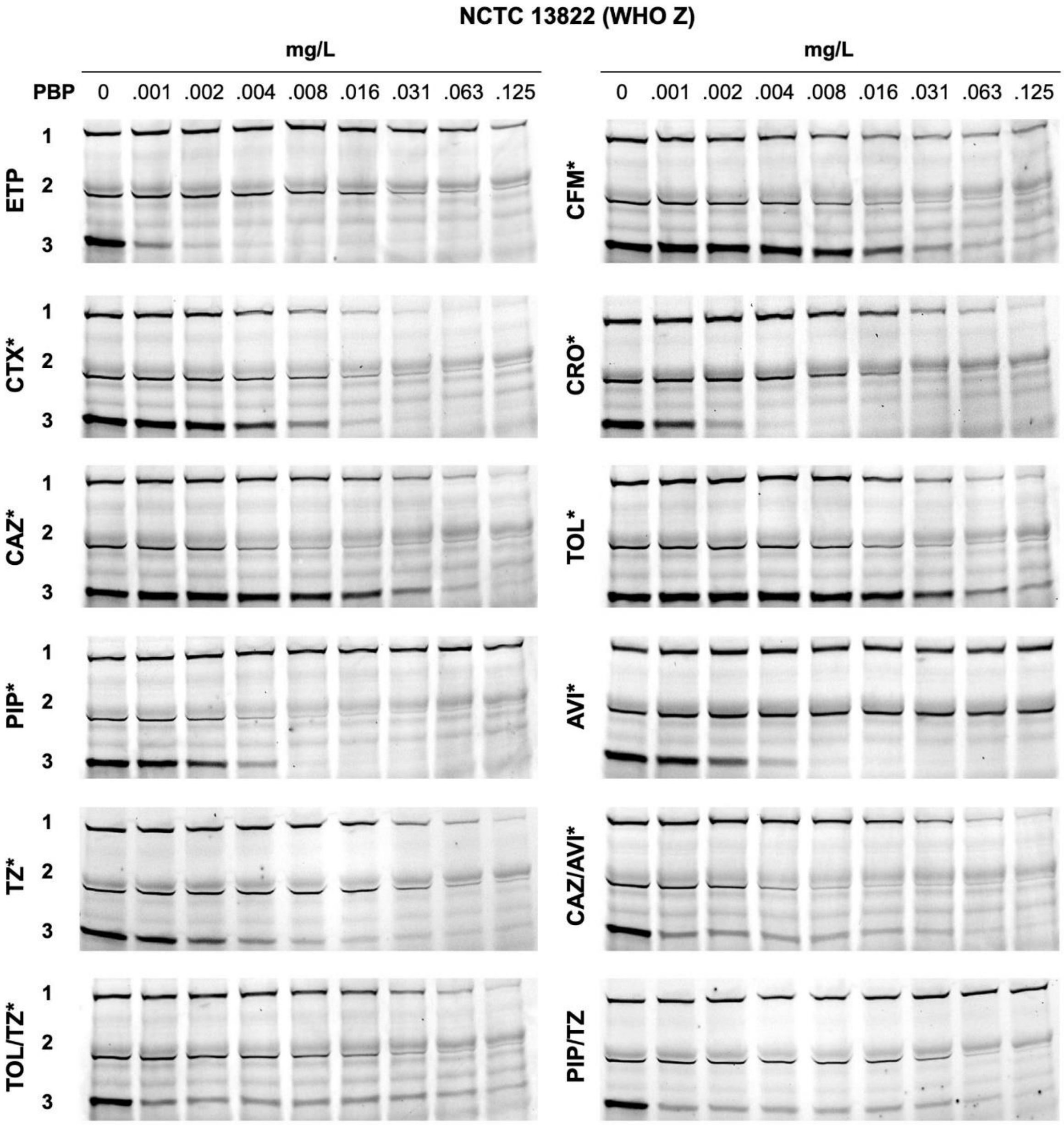

*CFM, 0.25-32 mg/L; CTX, 0.125-16 mg/L; CRO, 0.016-2 mg/L; CAZ, 0.25-32 mg/L; TOL, 1-128 mg/L; PIP, 0.016-2 mg/L; AVI, 2-256 mg/L; TZ, 1-128 mg/L; CAZ/AVI, 0.25-32 mg/L (avibactam 4 mg/L fixed concentration); TOL/TZ, 1-128 mg/L (tazobactam 4 mg/L fixed concentration).

PBP-binding assay gels for *N. gonorrhoeae* strains ATCC 19424, ATCC 49226; clinical strains NG 3, NG 7, NG 12, NG 14, NG 19, NG 20, NG 21 and NG 22; WHO reference strains NCTC 13820 (WHO X), NCTC 13821 (WHO Y) and NCTC 13822 (WHO Z). [1] Drugs tested were ertapenem (ETP), cefixime (CFM), cefotaxime (CTX), ceftriaxone (CRO), ceftazidime (CAZ), ceftolozane (TOL), piperacillin (PIP), avibactam (AVI), tazobactam (TZ), ceftazidime/avibactam (CAZ/AVI), ceftolozane/tazobactam (TOL/TZ), and piperacillin/tazobactam (PIP/TZ). The antibiotic-bound PBP-containing membrane

preparations were label with 25 μM Bocillin FL^{TM}. Labeled PBPs were separated by SDS-PAGE and detected using a fluorimager. The global range of concentrations tested was 0.001 to 512 mg/L.

REFERENCES

1. Unemo, M., et al., *The novel 2016 WHO Neisseria gonorrhoeae reference strains for global quality assurance of laboratory investigations: phenotypic, genetic and reference genome characterization.* J Antimicrob Chemother, 2016. **71**(11): p. 3096-3108.
