# Supplementary material for: Differential contribution of PBP occupancy and efflux on the effectiveness of β-lactams at their target site in clinical isolates of Neisseria gonorrhoeae
Source: PLoS Pathog. 2024 Dec 31;20(12):e1012783. doi: 10.1371/journal.ppat.1012783 (PMC11729944; doi:10.1371/journal.ppat.1012783)
Supplement: Attachment — Submitted filename: Reviewer_Comments.pdf [file ppat.1012783.s007.pdf]

Reviewer's Responses to Questions

Part I - Summary
Please use this section to discuss strengths/weaknesses of study, novelty/significance, general execution and scholarship.

Reviewer #1: This paper rigorously shows the impact of certain beta-lactams on the major PBPs of Neisseria gonorrhoeae, the influence of a multi-drug efflux pump system on resistance to multiple antibiotics in this class as well as the efficacy of potential combination drugs. The work was carefully performed, and the data seem solid. A minor weakness is the lack of establishing that the efflux pump inhibitors that were used can produce a phenotype similar to what is observed with an efflux pump null mutant.

Reviewer #2: This study investigates the relationship between PBP alleles and susceptibility to antibiotics used to treat gonorrhoeae, including last line cephalosporins. By carrying out multiple MIC assays with different drug combination, the authors identified the importance of PBP alleles in resistance against beta lactams and cephalosporin, and showed that inhibiting efflux pumps decreased the MICs of these antibiotics approx. 10-fold (or less), which is in line with previous studies.

Additionally, the authors have done a lot of work isolating PBPs to identify relationships between the biochemical relationship between PBP alleles and their affinities with different drugs and drug combinations, with the MICs of different antibiotics. There have been previous related studies establishing the link between different PBP alleles and beta lactam derivatives; despite that, this study stands out in two ways: firstly, it provides biochemical support with PBPs and the corresponding drug targets, rather than relying only on a correlation between PBP alleles observed and MIC values observed. Secondly, the study focuses on evaluating the effectiveness of the few remaining antibiotics that can be used to treat gonorrhoeae and is therefore potentially clinically important.

We express our gratitude to the reviewers and the editor for their thorough evaluation and insightful feedback on our research.

Part II – Major Issues: Key Experiments Required for Acceptance
Please use this section to detail the key new experiments or modifications of existing experiments that should be absolutely required to validate study conclusions.

Generally, there should be no more than 3 such required experiments or major modifications for a "Major Revision" recommendation. If more than 3 experiments

are necessary to validate the study conclusions, then you are encouraged to recommend "Reject".

Reviewer #1: The work would be considerably strengthened if the authors used an mtrD null mutant instead of efflux pump inhibitors in their work. Without this, it is not really possible to correlate their findings with that of Golparian et al, which they reference. Furthermore, the authors should include penicillin in their work on the impact of the MtrCDE efflux pump. If they can get the same result as as Golparian et al obtained with strain H041 vs H041 mtrD::kan then i would be satisfied.

We have successfully inactivated the *mtrD* gene in our selected strains through two methodologies, constructing pUCP*mtrD*::Km vector and utilizing DNA derived from the KH14 strain, as referenced in Golparian et al. 2014 and various studies conducted by Shafer et al. Additionally, penicillin G was incorporated into our experimental framework. The results indicated that strain H41 (WHOX) exhibited consistent levels of minimum inhibitory concentration (MIC) reduction across all tested compounds, aligning with the findings of Golparian et al. 2014. While the addition of an efflux pump inhibitor resulted in comparable MIC values, the inactivation of *mtrD* generally produced lower MIC values for cephalosporins and piperacillin.

One comment that is of interest: ertapenem was used to treat the patient in England with an MDR gonorrheal infection in 2016. This is an expensive antibiotic and likely cost-prohibitive for low-middle income countries. In addition, IV treatment required extensive clinical management.

We fully concur with the reviewer's observations concerning ertapenem. While we acknowledge that it is not a universal solution, it may indeed serve as a viable option in specific instances of complicated gonorrhea caused by ESC-resistant strains.

Reviewer #2: The authors established a link between PBP IC50 and MICs using only eight strains; however, these PBP alleles were never cloned into a standard strain to determine their importance in resistance of the antibiotic agents tested. It would be highly beneficial to either 1) investigate other isolates with PBP2 alleles identical to those which were tested and determine what these isolates' MICs of the antibiotics tested are to further validate the conclusions on a global population scale, or 2) clone these into eg. FA1090 and measure changes in MIC to confirm that the PBP alleles described are indeed responsible for changes in MICs (with reference to Figs 2 and 3).

We have cloned the PBP2 alleles of the WHO ESC-resistant strains and the majority of our clinical isolates in accordance with the reviewer's suggestions. Because of their low β-lactam MICs, transformants of *penA* variants from isolates NG7, NG19, and NG20 were not recoverable. Our findings were comparable to those reported

by Unemo et al. 2012 and Ohnishi et al. 2011, about the transformation of *penA* ESC-resistant mosaic variants (with cephalosporin MICs 125–500 times higher).

Part III – Minor Issues: Editorial and Data Presentation Modifications
Please use this section for editorial suggestions as well as relatively minor modifications of existing data that would enhance clarity.

Reviewer #1: There are a number of minor editorial issues that need to be corrected.
1. Line 63: more recent estimates indicate that there is ca. 82 million cases of gonorrhea worldwide/year.

The text has been changed accordingly.

2. Line 100: The mtrR and mtrCDE promoters are not shared but overlap on different strands at the -35 hexamer. The paper by Hagman et al, 1995 should be referenced.

Text has been changed and the reference has been added.

3. Line 119: insert the word "above" after "consistently"

The sentence has been changed for added clarity.

4. Line 185: insert the word "of" after "any"

“Of” has been inserted.

5. Line 328: there seems to be a word missing after: ceftriaxone and....

The word “and” has been deleted.

6. Some references are incomplete-see #57 as an example

The references have been checked and corrected accordingly.

7. Figure 3 is very confusing as the legend indicates that multiple strains were studied but the figure seems to be the result of 1 strain with multiple antibiotics.

Scatter plots were employed to illustrate the correlation between PBP2 IC_{50} values and the minimum inhibitory concentrations (MIC) of β-lactam antibiotics, β-lactamase inhibitors (BLI), and the combination of β-lactams with BLI. Each plot

depicts the relationship between PBP2 IC50 and the MICs for each drug across all tested strains.

Reviewer #2: -Please italicize gene names (e.g. penA in line 97)

Gene names have been italicized throughout the manuscript.

-Line 243: "Said isolates" - unclear which isolates these said isolates referred to. I suppose these are the resistant ones?
The sentence has been rewritten for clarity.

-Line 271: "This is led" - no need for is

Has been changed accordingly.

-Consider including the Chen et al., 2019 paper on decreasing mtrCDE efflux pump expression resulting in increasing gonococcal susceptibility to beta lactam in the discussion section on the contribution of efflux pump to beta lactam MICs. This will help situate the finding compared to other papers in general.

According to the reviewer’s recommendation, the above mentioned reference has been added.

-The phylogenetic tree was used to point out the penA alleles and other resistance determinants; it would be clearer instead to just show the typing, genomic resistance and phenotypic resistance, as no further reference is made to the phylogenetic relationship between the strains in the paper. However, if a phylogenetic tree is desired, the tree constructed needs to take into account homologous recombination between N. gonorrhoeae - it is unclear whether this was considered in the methods section. Alternatively, GrapeTree in PubMLST could be used to examine genomic relationships between the eight isolates.

Phylogenetic tree has been deleted from the figure.
